# Factor-driven urban sensory equity: parallel auditory and olfactory perceptual models of spatial experience in urban environments for people with visual impairment

**DOI:** 10.3389/fpsyg.2026.1784190

**Published:** 2026-03-09

**Authors:** Meihui Ba, Jingyu Fang, Zhongzhe Li, Weihang Xu, Jian Kang

**Affiliations:** 1 Pan Tianshou College of Architecture, Art, and Design, Ningbo University, Ningbo, China; 2 Institute for Environmental Design and Engineering, The Bartlett, University College London (UCL), London, United Kingdom

**Keywords:** auditory, olfactory, perceptual model, spatial experience, urban environment, visually impaired

## Abstract

Visually impaired people constitute a large proportion of the population, and audition and olfaction serve as vital media through which they perceive urban environments. However, few studies have focused on parallel auditory and olfactory perceptual models of visually impaired individuals to comprehensively describe how they understand the environment, thus providing evidence-based urban spatial design interventions. This study aimed to construct a parallel auditory and olfactory perceptual model of the visually impaired in urban environments to characterise perceptual dimensions of spatial experience and explore the relationships among different perceptual factors. First, indicators of auditory and olfactory environmental perceptions specific to the visually impaired were extracted through interviews. Subsequently, a laboratory experiment was conducted to investigate the evaluations of the visually impaired (participants were adults with visual impairment defined as best-corrected visual acuity below 0.1) under different combinations of auditory and olfactory variables, based on which a perceptual model was constructed. This study identified 17 indicators of auditory environment perception and 10 indicators of olfactory environment perception through interviews. Through factor analysis, these indicators were grouped into five perceptual factors of the auditory environment (auditory affect, auditory spatiotemporality, auditory discriminability, auditory awareness, and auditory curiosity) and three perceptual factors of the olfactory environment (olfactory affect, olfactory awareness, and olfactory spatiality). The distributions of auditory and olfactory environment perceptions under different auditory and olfactory variables were analysed. Furthermore, the correlations between these indicators were examined to assess the feasibility of simplifying the indicator framework. This study provides a practical, evidence-based guideline, which offer tangible utility for the researchers and practitioners in urban environmental design.

## 1 Introduction

For Globally, safeguarding the livelihoods and rights of persons with disabilities is of significant importance (Hendriks, 2007). At present, there are at least 2.2 billion visually impaired people (World Health Organization, 2019), with the number exhibiting a rising trend every year. Unlike individuals with other types of disabilities, those with visual impairments face distinct challenges in spatial and environmental experience owing to limitations in visual information. They often struggle to perceive detailed information and dynamic changes within complex environments in a timely manner (Lin et al., 2025). Therefore, individuals with visual impairments tend to rely on their heightened senses to acquire environmental information and utilise cross-modal abilities to compensate for missing inputs, resulting in their non-visual senses being more sensitive than those of sighted individuals (Dischinger, 2000). Audition and olfaction become important mediators in the process by which visually impaired individuals form an overall perception of urban environments (touch and taste are active modalities that rely more on subjective intentions) (Ba and Kang, 2019). Auditory and olfactory factors constitute the core information for spatial experience in visually impaired individuals. However, owing to inadequate consideration of their needs in contemporary urban and architectural space design, individuals with visual impairment remain scarcely visible in daily life despite their substantial population base. This leads to gradual detachment from society, which adversely affects physical and mental well-being. Therefore, the targeted integrative design of urban auditory and olfactory environments constitutes a crucial approach supporting spatial experience in visually impaired individuals and promoting urban inclusivity (Ba et al., 2023).

Spatial experience refers to the experiences that enable individuals to represent, update, and use information about spatial layouts and one’s position within them, supporting functions such as orientation, route learning, landmark/place identification, and spatial memory (Denis, 2017; Newcombe, 2004). For people with visual impairment, non-visual cues become especially important: auditory information can support distance estimation, direction finding, and scene recognition, whereas olfactory cues may act as place markers and memory triggers that help confirm location and guide wayfinding when sources are relatively stable in space (Koutsoklenis and Papadopoulos, 2011; Feng et al., 2019). Related work on sensory memory further suggests that multisensory traces can be “spatialised,” reinforcing links between smell, sound, and remembered place experience (Xiao and Lindborg, 2025). Studies have also found that visually impaired people tend to categorise environments into two types: safe and secure, or harmful and dangerous. The home is perceived as safe, whereas environments outside it are often considered hazardous. Consequently, safety is a fundamental requirement for their spatial experience (Imrie and Kumar, 1998). Moreover, the perceived safety of the environment is more important than subjective preference (Mediastika et al., 2020), and this perception of safety is primarily derived from auditory perception (Papadopoulos et al., 2012). Furthermore, studies have investigated the experiences of the visually impaired within architectures using virtual reality technology, indicating that the auditory environment can facilitate the formation of cognitive maps of spaces; however, an excess of auditory stimuli may impose a cognitive burden (May et al., 2020). A study based on the concept of shared spaces surveyed visually impaired individuals and found that orientation difficulties during spatial use constitute a major challenge, while auditory ability is closely related to spatial positioning (Carlson–Smith and Wiener, 1996). Therefore, it is necessary to provide them with a rich sensory environment, particularly to meet their navigational needs, by enhancing their auditory and other sensory cues (Havik et al., 2015; Parkin and Smithies, 2012). In terms of olfactory perception in urban environments, vision and audition are generally regarded as sources of knowledge, whereas olfaction is often associated with emotion and memory (Feng et al., 2019), and is commonly considered less important than the former. However, studies have found that visually impaired individuals rely on olfaction to identify objects or spaces and construct both personal and collective identities (Ghosh, 2022). Other research has focused on the most commonly used olfactory cues and their usage patterns among visually impaired individuals and identified the significance of different olfactory cues for wayfinding in urban settings (Koutsoklenis and Papadopoulos, 2011). On this basis, emotional needs such as comfort in the auditory and olfactory environments are considered as further needs that arise following the fulfilment of fundamental requirements such as safety and navigability (Li et al., 2024) and are equally worthy of attention. Studies on the perceived restorativeness of parks by the visually impaired have shown that their evaluations are lower than those of sighted individuals (Boucherit et al., 2023), indicating that visually impaired individuals may have higher demands for restorative environments. In terms of the types of urban environments, most studies on the perception of the visually impaired have focused on parks and gardens (Mediastica et al., 2019; Zajadacz and Lubarska, 2020), as these contain abundant non-visual sensory elements. Research has also shown that owing to the limited range of activities of the visually impaired, community public spaces often serve as the primary venues for their activities and social interactions (Ba et al., 2023), and the quality of these environments greatly influences the lifestyle of visually impaired populations. Therefore, creating community public spaces suitable for the perception and use of visually impaired individuals is an important component of enhancing urban health performance and promoting health equity (Yang et al., 2018).

A rational measure and comprehensive description of the perceptions of auditory and olfactory environments that facilitate spatial experience for visually impaired individuals, as well as the construction of a perceptual model suitable for them, form the foundation for a thorough understanding of their needs, thereby enabling a more targeted practical design. However, existing perceptual-model research has largely focused on auditory environments and sighted populations, while still offering useful insights that can be adapted to visually impaired users. Regarding the auditory perception of urban environments, one study investigated the perception of visually impaired in park soundscape, aiming to identify their unique soundscape perceptual dimensions elicited solely through auditory cues. The findings revealed that these unique dimensions include danger, direction, space, and nature (Mediastika et al., 2020). Existing research defines the perceptual dimensions of affective responses to auditory environments in outdoor spaces using listening experiments (Axelsson et al., 2010). This model has been adopted by ISO TS 12913-3, with its core components used as descriptors on a five-point scale, becoming standardised indicators that are widely applied in the evaluation of urban outdoor soundscapes (International Organization for Standardization, 2019). Similarly, some studies have proposed perceptual dimensions of the auditory environment from the perspective of indoor spaces, and these models are mostly applied to the evaluation of urban indoor soundscapes (Torresin et al., 2020; Torresin et al., 2023). In terms of olfactory perception in urban environments, through on-site urban smellwalks combined with interviews, a perceptual model of urban smellscapes for sighted individuals centred on pleasantness was developed and nine indicators influencing pleasantness were extracted from the interviews (Xiao et al., 2018). Study on indoor smellscapes targeting sighted individuals has shown that pleasantness, presence, and naturalness are the three main perceptual dimensions (Torriani et al., 2025). Compared with the auditory environments, perceptual models of olfactory environments remain less developed and less frequently applied in environmental design research, despite growing recognition that odours contribute to place meaning, comfort, and behavioural tendencies. Importantly, both strands of modelling have predominantly been established using sighted or general populations, leaving open the question of whether—and how—auditory and olfactory perceptual dimensions differ for people with visual impairment, for whom non-visual cues are central to everyday environmental understanding.

This gap motivates the present study, which derives evaluation indicators from people with visual impairment and examines the latent perceptual dimensions of auditory and olfactory environments to inform inclusive multisensory design in community public spaces. Based on this, this study employed a mixed-methods research approach that integrated qualitative and quantitative methodologies. Initially, interviews were conducted to capture the experiences of visually impaired individuals regarding urban auditory and olfactory environments, with the aim of elucidating the key concerns they associate with these environments that contribute to spatial experience through self-descriptions. Subsequently, a laboratory experiment was conducted to examine the perceptual evaluations of the visually impaired under different combinations of auditory and olfactory factors, based on which a parallel auditory and olfactory perceptual model was developed to promote spatial experience. The specific research questions addressed in this study are as follows: (1) Which auditory and olfactory evaluation indicators do people with visual impairment consider salient in urban environments from semi-structured interviews? (2) How do these indicators organise into underlying perceptual dimensions of the auditory environment under controlled laboratory sound conditions? (3) How do these indicators organise into underlying perceptual dimensions of the olfactory environment under controlled laboratory odour conditions? In addition, the Discussions examines which perceptual dimensions are shared across auditory and olfactory environment perceptions and which are modality-specific. The findings of this study will have important implications for the development of spatial cognitive rehabilitation training strategies for visually impaired individuals and the design of accessible urban environments.

## 2 Materials and methods

### 2.1 Study design

**Figure 1:**
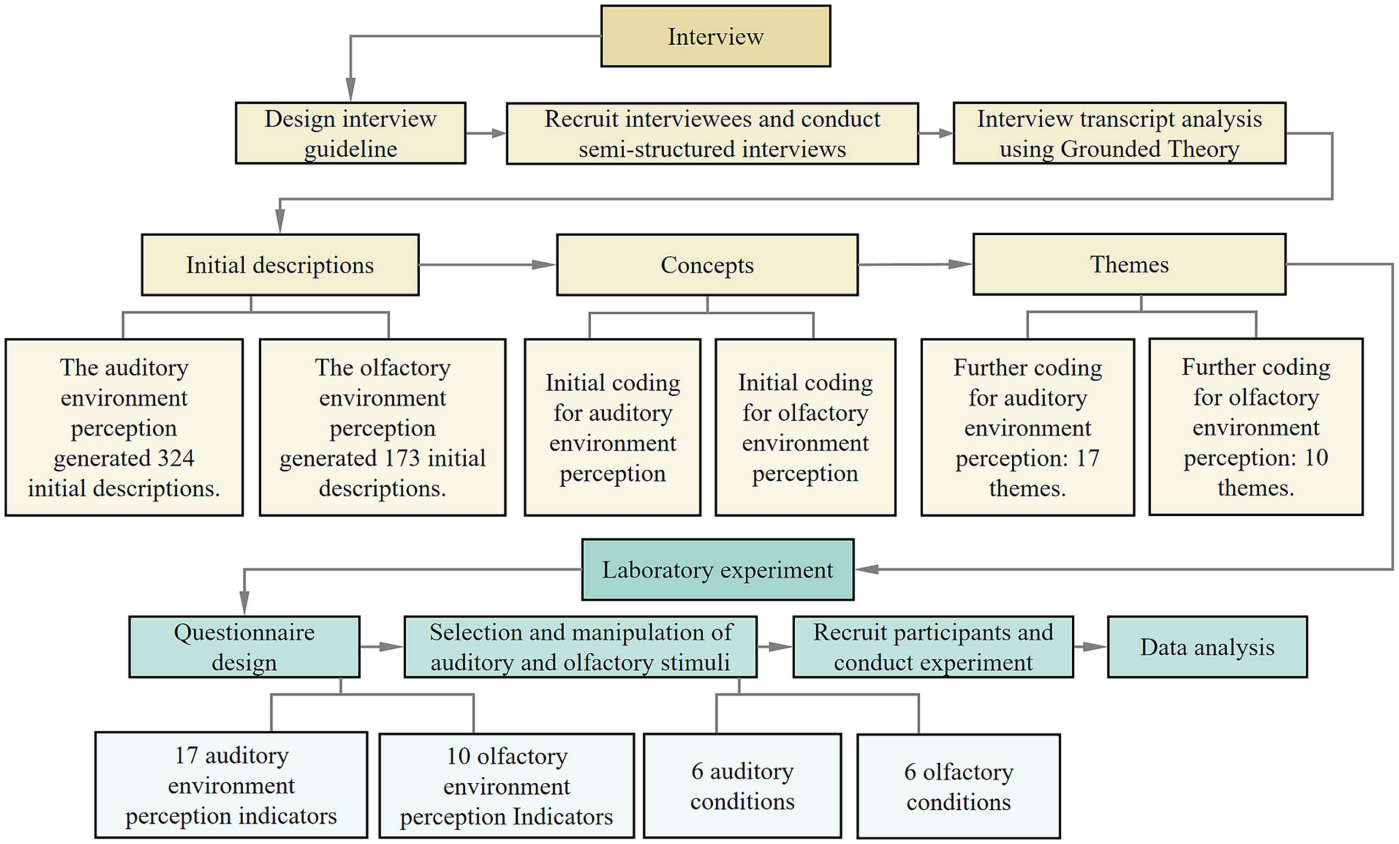
Research framework.

Semi-structured interviews were conducted to explore the auditory and olfactory experiences of visually impaired individuals in urban community public spaces. Using a grounded-theory-informed inductive coding approach, this study derived evaluation indicators from participants’ own descriptions in a context where established indicator sets for visually impaired people whose auditory–olfactory experience remain limited. And the aim was indicator generation for subsequent quantitative testing, rather than developing a full grounded theory. Subsequently, a laboratory experiment was conducted to obtain evaluation results of the previously extracted indicators in different auditory-olfactory interactive environments. Factor analysis was then employed to reduce the dimensionality of these evaluation indicators, by aiming to establish a parallel auditory and olfactory perceptual model for the visually impaired to simplify multiple indicators. The research framework is illustrated in Figure 1.

### 2.2 Interview

#### 2.2.1 Interview design

To gain a comprehensive understanding of how the visually impaired perceive the auditory and olfactory environments of urban spaces, this study employed semi-structured interviews (Jia et al., 2020). Because of the limited range of activities of the visually impaired, community public spaces frequently serve as primary venues for their daily activities and social interactions (Ba et al., 2023; Li et al., 2024). Therefore, to ensure that the interviews remained focused, the context was defined as community public space. According to the research aim, the interview consisted of three parts: (1) introductory questions: this involved interviewees’ attitudes towards community public spaces, serving as a lead-in to the themes of auditory and olfactory perception. (2) Key questions: this involved interviewees’ perceptions of auditory and olfactory environments and their reasons. Auditory and olfactory factors usually appear simultaneously in the environment; thus, the auditory-olfactory interactive environment was intended to determine whether visually impaired individuals had other specific perceptions in complex environments. Moreover, these three parts encouraged interviewees to exhaust all possible responses until no new answers were provided. (3) Ending questions: these involved supplementary opinions and interviewees’ personal information to avoid information loss and to explore the reasons for specific results. Table 1 presents the guideline for semi-structured interviews. This paper uses “supporting spatial experience” to refer specifically to perceptual dimensions relevant to orientation and distance judgement, place/scene identification, comfort and experiential qualities when using space, etc. Each interview lasted approximately 40–60 min and was audio-recorded for subsequent analysis.

**Table 1:** Guideline for semi-structured interview.

Category		Questions
Introductory questions		Do you usually go out for activities? Where do you usually go for activities? How often do you participate in these activities? Which factors in community public spaces are most important to you in forming an overall spatial impression? What specific benefits do these factors provide you?
Key questions	Auditory environment	4. What sounds can you hear in the community public spaces you described? 5. What kind of feelings does the auditory environment there evoke for you? Why do you have these feelings? 6. How do these sounds you mentioned affect or assist your spatial experience? Why?
	Olfactory environment	7. What odours can you smell in the community public spaces you described? 8. What kind of feelings does the olfactory environment there evoke for you? Why do you have these feelings? 9. How do the odours you mentioned affect or assist your spatial experience? Why?
	Auditory-olfactory interactive environment	10. What sounds and odours simultaneously occur in the community public spaces you described? 11. What kind of feelings does the auditory-olfactory interactive environment there evoke for you? Why do you have these feelings? 12. How do the combinations of sounds and odours you mentioned affect or assist your spatial experience? Why?
Ending questions		13. Do you have any additional comments?
		14. Your personal informations (sex, age, degree of visual impairment, congenital or acquired visual impairment, education level, and occupation)

#### 2.2.2 Interviewees

This study recruited 25 visually impaired individuals (aged 18–60; 12 females, 13 males) via online interviews. All interviewees had a best-corrected visual acuity below 0.1 (19 were totally blind and six had low vision). The number of interviewees was determined based on the consideration that adding more samples would no longer yield additional new answers. During the interviews, the researcher explained the study’s aim and procedure in clear and accessible language and ensured strict confidentiality of the participants’ personal information.

#### 2.2.3 Grounded theory

The interview recordings were transcribed verbatim. Then grounded-theory-informed procedures were applied to generate evaluation indicators (Xiao et al., 2018; Jia et al., 2020), following constant comparison across transcribed meaningful units were labelled using participants’ original wording (e.g., “feeling comfortable,” “helping to estimate the time of day”). Second, labels were compared and merged into higher-level concepts (initial coding; e.g., “pleasant,” “joyful”). Third, conceptually similar concepts were grouped into themes (further coding) that served as candidate indicators (e.g., “pleasantness”). Coding continued until no substantively new themes emerged.

### 2.3 Laboratory experiment

#### 2.3.1 Questionnaire design

The questionnaire for the laboratory experiment was designed based on themes derived from the grounded theory analysis (see Section 3.1 for interview results). The questionnaire consisted of two parts: an evaluation of the auditory and olfactory environments. A 5-point Likert scale was used, where, for instance, familiarity was rated as follows: 1–5 represented very unfamiliar, unfamiliar, neutral, familiar, and very familiar, respectively. The specific evaluation indicators and questions are listed in Table 2. At the end of the questionnaire, participants were asked personal questions regarding their sex, age, visual impairment status, and place of residence.

**Table 2:** Questionnaire of auditory and olfactory environments.

Category	Interview-derived themes	Questions
Auditory environment	Pleasantness	Does the current auditory environment provide a sense of pleasantness?
	Comfort	Does the current auditory environment provide a sense of comfort?
	Safety	Does the current auditory environment provide a sense of safety?
	Familiarity	Does the current auditory environment provide a sense of familiarity?
	Curiosity	Does the current auditory environment provide a sense of curiosity?
	Annoyance	Does the current auditory environment provide a sense of annoyance?
	Fear	Does the current auditory environment provide a sense of fear?
	Recognition	Does the current auditory environment provide a sense of recognition?
	Time	Does the current auditory environment provide a sense of time?
	Loudness	How would you rate the loudness of the current auditory environment?
	Warning	Does the current auditory environment provide a sense of warning?
	Reverberation	Does the current auditory environment provide a sense of reverberation?
	Interference	Does the current auditory environment provide a sense of interference?
	Guidance	Does the current auditory environment provide a sense of guidance?
	Distance	Can you assess the distance between yourself and the sound source?
	Orientation	Can you determine the direction of the sound source?
	Scale	Can you assess the scale of the space from which the sound comes?
Olfactory environment	Pleasantness	Does the current olfactory environment provide a sense of pleasantness?
	Comfort	Does the current olfactory environment provide a sense of comfort?
	Freshness	Does the current olfactory environment provide a sense of freshness?
	Familiarity	Does the current olfactory environment provide a sense of familiarity?
	Recollection	Does the current olfactory environment provide a sense of recollection?
	Recognition	Does the current olfactory environment provide a sense of recognition?
	Intensity	How would you rate the intensity of the current olfactory environment?
	Warning	Does the current olfactory environment provide a sense of warning?
	Distance	Can you assess the distance between yourself and the odour source?
	Orientation	Can you determine the direction of the odour source?

#### 2.3.2 Selection and manipulation of auditory and olfactory stimuli

This study simulated an auditory-olfactory interactive environment by presenting typical sounds and odours from community public spaces in a laboratory setting. The sounds and odours were selected based on the interview results, with the following selection criteria: (1) the sound or odour was mentioned by more than half of the visually impaired interviewees, representing its perceptual commonality; (2) the sounds included the three typical categories of sound sources in urban soundscapes: natural sounds, anthropogenic sounds, and mechanical sounds (Kang, 2006); the odours included the typical odour sources of urban smellscapes: natural odours, artificial odours, and industrial odours (Henshaw, 2013); (3) the sounds and odours had to be both accessible and feasible for laboratory simulation and non-harmful to human health. Ultimately, five types of sounds were selected: traffic, foliage, conversation, bird, and music, along with five types of odours: grass, soil, hot pot, osmanthus, and coffee. In addition, blank control conditions were included, in which the sound was an ambient background sound from an open community space without a dominant sound source, and the odour was without stimuli.

For the manipulation of the auditory stimuli, a four-channel portable acquisition front-end and mobile recording and playback system (SQobold) was used. Field recordings were conducted in urban public spaces with selected dominant sound sources and without other interfering noises. For background sounds without a dominant sound source, the recording was conducted in urban public open spaces at midnight. The equipment was positioned at a height of 1.5 metres above the ground during recording (Zahorik, 2002). Each audio clip was recorded for at least 5 min. The recordings were processed for sound pressure level adjustment and edited using the Adobe Audition software. Each recording was adjusted to 50 dB, as this level is clearly audible in a laboratory setting without being overly loud (Jia et al., 2020), and was then trimmed to 2 min in length. The processed sound stimuli were played to the participants using high-fidelity headphones (WH-CH720N).

To manipulate the olfactory stimuli, essential oils or perfumes were used, with brands selected based on their high ratings of perceived authenticity (Ba and Kang, 2019). Two perfumes (osmanthus and grass) and three essential oils (hot pot, coffee, and soil) that passed authenticity tests were chosen. To control the odour intensity, distilled water was added to the odour materials to adjust their concentration. Precise odourant delivery was achieved via graduated syringe deposition, ensuring a uniform coating on five medical-grade cotton balls housed in a standardised 200-ml beaker. Prior to the formal experiment, five concentration gradients were prepared for each odour and 30 participants were invited to evaluate the subjective intensity. The sample concentration rated as moderate was selected for use in the formal experiments (Ba and Kang, 2019). Owing to differences in the diffusion rates of various odour source materials, participants could not perceive each given odour simultaneously after the beaker lid was opened. Therefore, during the preliminary study, the time interval between opening the lid and the participant’s first detection of each odour was recorded. The average time across all the participants was then used to determine the time at which the lid should be opened in the formal experiment to ensure that the auditory and olfactory stimuli could be presented simultaneously. According to the measurement results, osmanthus, soil, hot pot, coffee, and grass odours became perceivable approximately 10s, 5 s, 5 s, 5 s, and 10s after opening the lid, respectively.

#### 2.3.3 Participants

A total of 36 visually impaired participants (aged 20–70 years, with a sex ratio of 1:1) were recruited offline for the experiment. All participants had a best-corrected visual acuity below 0.1 (30 were completely blind and six had low vision). All the participants self-reported normal audition and olfaction. On the day of the experiment, none of the participants wore perfumes and there were no smokers or other factors that could have affected the experimental results. Ethical approval for this study was granted by authors’ affiliation, all procedures were performed in compliance with relevant laws and institutional guidelines, and the informed consent was obtained for experimentation.

#### 2.3.4 Experimental procedure

**Figure 2:**
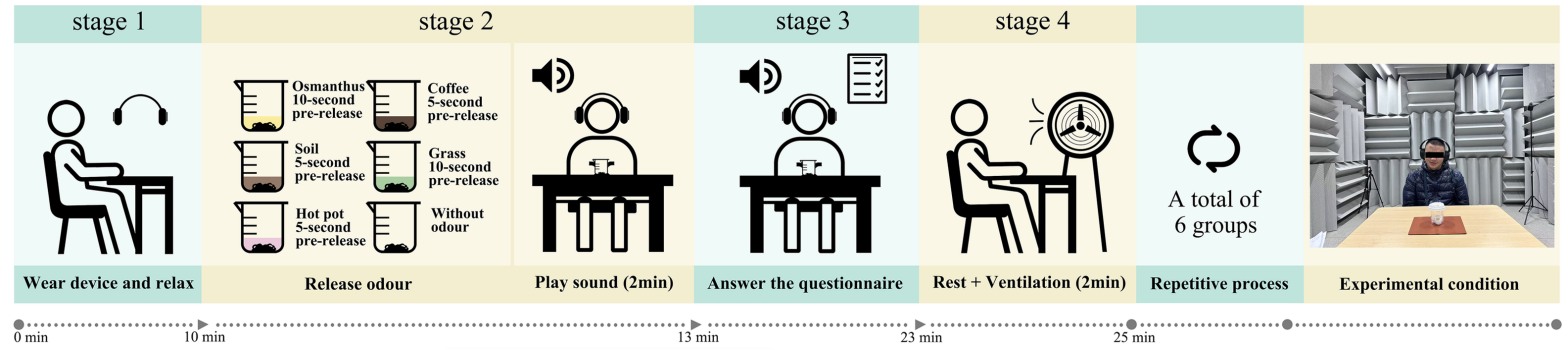
Experimental process flow diagram.

The experiment was conducted in a semi-anechoic chamber with a background noise level of 20 dB. The experiment was conducted on a one-on-one single-session basis. Before starting, visually impaired participants were given 5 min to acclimate to the laboratory environment to alleviate any discomfort or fear. Subsequently, the researcher explained the experimental requirements and relevant precautions to ensure that the participants fully understood the aim and procedures of the experiment. Each participant was randomly presented with six combinations of auditory and olfactory stimuli. Specifically, a fully crossed sound × odour design was employed: six sound types and six odour types (including background sound/without odour condition) were combined to form 36 sound–odour pairings (6 × 6). Each participant completed six trials, with one pairing presented per trial, and the sound types, odour types, and their pairings were evenly distributed across serial positions. The full trial-order schedule is provided in Supplementary Table 1. Prior to playing the sounds, the lids of the beakers containing the odour materials were opened according to the pre-calculated times for each odour to become perceivable. During odour presentation, the participant’s nose was maintained at a standard distance of 45 cm from the odour source. After the stimuli presentation, participants were given 30 s to experience it, followed by a questionnaire session, during which the stimuli continued to be presented. The researcher read the questionnaire verbatim to the participants, who rated them according to the questions, while the researcher recorded their responses. After each condition, there was a 2-min rest period, during which the laboratory was ventilated using a ventilation duct connected to a fan with an airflow rate of 8,700 m^3^/h. The experimental process is illustrated in Figure 2. The duration of each participant’s experiment was approximately 40 min.

#### 2.3.5 Data analysis

This study conducted a factor analysis of the perception of auditory and olfactory environments, aiming to reduce the dimensionality of multiple indicators extracted from the interviews and to analyse the relationships between different factors. Exploratory factor analysis (EFA) was conducted in SPSS separately for the auditory and olfactory item sets. Each participant completed six sound–odour trials, and each trial contributed one row of item ratings; therefore, the input matrix comprised 216 rows (36 × 6), and ratings were not aggregated to participant. The number of retained factors was determined primarily using the Kaiser criterion (eigenvalues > 1) together with inspection of the scree plot; varimax orthogonal rotation (with Kaiser normalisation) was applied to obtain interpretable and relatively independent dimensions for subsequent two-dimensional mapping. Eigenvalues, percentage variance explained, and cumulative variance are summarised in Supplementary Table 2. Subsequently, two-dimensional distributions were used to present the spatial distribution of auditory and olfactory variables in the factor analysis. This approach not only illustrates variations in the evaluations of individual auditory or olfactory variables, including boundary shapes and dispersion levels, but also demonstrates more clearly the similarities among different auditory or olfactory variables. All distribution areas were represented using the 50th percentile contour. Finally, Spearman correlation analyses were performed separately on indicators of auditory and olfactory environment perceptions, with the aim of assessing whether highly correlated indicators could be fitted together to streamline them.

## 3 Results

### 3.1 Indicators of auditory and olfactory environment perceptions

The coding framework for the interview results on auditory and olfactory environment perceptions is presented in Table 3. For auditory environment perception, 324 original descriptions (a selection is presented owing to the large volume) and 17 themes were extracted. For olfactory environment perception, 173 original descriptions and 10 themes were extracted. The themes identified from the interviews reflected the key concerns of environmental perception for visually impaired individuals in community public spaces and were thus extracted as the final evaluation indicators for auditory and olfactory environment perceptions.

**Table 3:** Coding frame of auditory and olfactory environment perceptions.

Category	Example of excerpts from the interview (original descriptions)	Concept (initial coding)	Theme (further coding)
Auditory	Sounds such as insect chirping and bird sounds give me a sense of being close to nature, which feels very pleasant Hearing the sounds of children also makes me feel joyful	Pleasant Joyful	Pleasantness
	I feel comfortable when I hear bird sounds The rustling of leaves makes me feel relaxed.	Comfortable relaxed	Comfort
	The sound of square dance is good because I believe that places with many people are safe If it is very quiet, I tend to feel more worried	Safe worried	Safety
	Hawking cry reminds me of familiar feelings from my childhood	Familiar	Familiarity
	Hawking cry makes me feel curious and eager to know what is being sold	Curious	Curiosity
	If I continuously hear car horns, I will feel very annoyed	Annoyed	Annoyance
	Hearing the sound of cars makes me feel scared	Scared	Fear
	Hearing the sound of a fountain makes me think there must be a small square ahead When I hear slow or dragging footsteps, I tend to think it’s an older person; fast footsteps make me think it’s a young person	Scene recognition Identity recognition	Recognition
	The level of traffic noise can give me a sense of the approximate time of day The sound of leaves is different in summer and autumn	Time season	Time
	I prefer this kind of quiet atmosphere and dislike overly noisy environments	Quiet	Loudness
	When I hear the sound of a car, I immediately become more alert to assess whether it poses any threat to my safety	Alertable	Warning
	In open spaces, no echo can be heard	Echo	Reverberation
	The sound of the wind can interfere with certain auditory cues from other sources.	Interfere	Interference
	Hearing the music played by someone running can guide me to follow behind them	Guide	Guidance
	Hearing a car horn can help me estimate the approximate distance	Distance	Distance
	Determining one’s position based on the orientation of a broadcast sound	Orientation	Orientation
	If I can hear two separate groups playing square dance music, I know the square is very large	Scale	Scale
Olfactory	Smelling the scent of grass makes me very pleasant	Pleasant	Pleasantness
	Smelling the fragrance of flowers is quite comfortable	Comfortable	Comfort
	The smell of fresh grass can be refreshing The odour of soil after rain is very fresh	Refreshing fresh	Freshness
	The smell of the soil just after the rain feels very familiar	Familiar	Familiarity
	The smell of fresh soil reminds me of my childhood	Recollection	Recollection
	The smell of the tree lets me know that there is a tree	Scene recognition	Recognition
	Some fragrance of flowers is very intense	Intense	Intensity
	The smell of the water indicates that there is a lake nearby, and I become more alert because I am afraid of falling into it	Alertable	Warning
	When I smell flowers, the intensity of their fragrance helps me determine how far they are from me	Distance	Distance
	When I smell garbage, I know there is a bin nearby, since its location is fixed, it helps me determine the orientation	Orientation	Orientation

### 3.2 Factors of auditory environment perception

**Figure 3:**
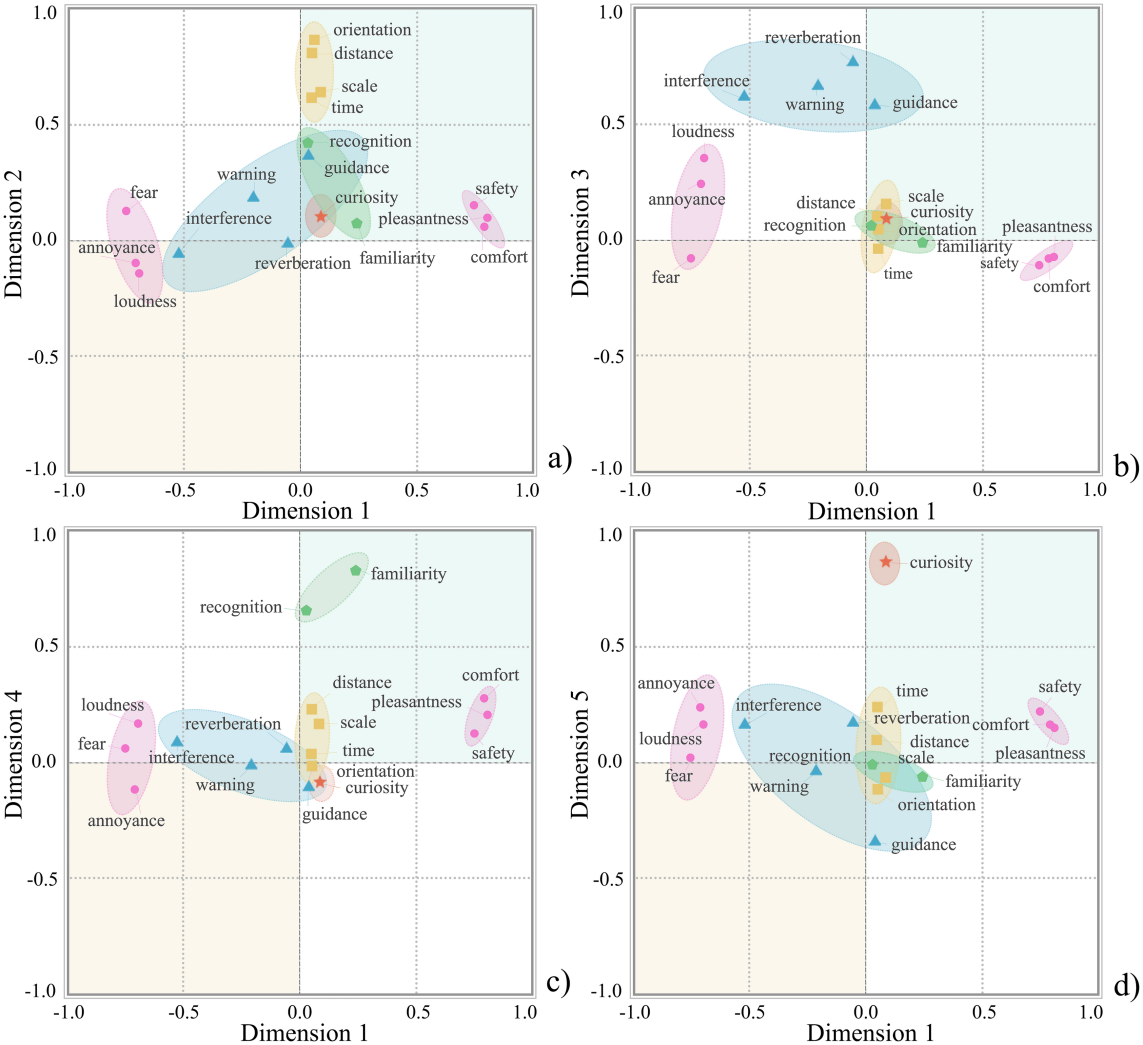
Factor loading plot of auditory environment perception. **(a)** Dimension 1 versus Dimension 2; **(b)** Dimension 1 versus Dimension 3; **(c)** Dimension 1 versus Dimension 4; **(d)** Dimension 1 versus Dimension 5.

Bartlett’s test of Sphericity for auditory environment perception was significant (*p* < 0.001) with a KMO value of 0.816, indicating that the data were suitable for factor analysis. The rotated factor matrix of auditory environment perception is shown in Table 4, and the factor loading plot of auditory environment perception is presented in Figure 3, in which different factors exhibit clear distributions across various dimensions. Factor 1 had high loadings on pleasantness, comfort, safety, annoyance, fear, and loudness and can be called auditory affect. Figure 3a shows that pleasantness, comfort, and safety are distributed along the positive axis of Dimension 1, whereas annoyance, fear, and loudness are distributed along the negative axis of Dimension 1. Factor 2 had high loadings on time, distance, orientation, and scale and can be referred to as auditory spatiotemporality. Figure 3a shows that these indicators are all distributed along the positive axis of Dimension 2. Factor 3 has high loadings on warning, reverberation, interference, and guidance and can be called auditory discriminability. Figure 3b shows that these indicators are all distributed along the positive axis of Dimension 3. Factor 4 had high loadings on familiarity and recognition and can be called auditory awareness. Figure 3c shows that these indicators are all distributed along the positive axis of Dimension 4. Factor 5 had a high loading on curiosity and can be called auditory curiosity. Notably, the Factor 5 was primarily defined by a single indicator. This pattern is linked to the indicator development: the evaluation indicators were translated from grounded-theory-derived themes, and certain theme is naturally operationalised as single indicator at the quantitative stage. Although this dimension is single-item, it showed a concentrated loading and clear separation from the other dimensions; therefore, it is retained and reported here as an independent dimension. Figure 3d shows that this indicator is distributed along the positive axis of Dimension 5.

**Table 4:** Rotated factor matrix of auditory environment perception.

Indicator	Factor				
	1	2	3	4	5
Pleasantness	**0.808**	0.095	−0.076	0.201	0.145
Comfort	**0.793**	0.056	−0.078	0.274	0.161
Safety	**0.748**	0.154	−0.107	0.119	0.217
Familiarity	0.242	0.075	−0.015	**0.827**	−0.071
Curiosity	0.087	0.102	0.092	−0.088	**0.863**
Annoyance	**−0.713**	−0.096	0.236	−0.120	0.238
Fear	**−0.752**	0.129	−0.077	0.057	0.023
Recognition	0.026	0.421	0.060	**0.651**	−0.016
Time	0.050	**0.616**	−0.038	0.035	0.239
Loudness	**−0.699**	−0.140	0.344	0.164	0.156
Warning	−0.206	0.184	**0.668**	−0.020	−0.039
Reverberation	−0.057	−0.013	**0.764**	0.057	0.166
Interference	−0.528	−0.061	**0.621**	0.084	0.156
Guidance	0.038	0.369	**0.584**	−0.113	−0.342
Distance	0.047	**0.810**	0.096	0.228	0.096
Orientation	0.056	**0.865**	0.051	−0.016	−0.122
Scale	0.084	**0.639**	0.156	0.160	−0.065

Bold indicates matrix loadings greater than 0.5.

**Figure 4:**
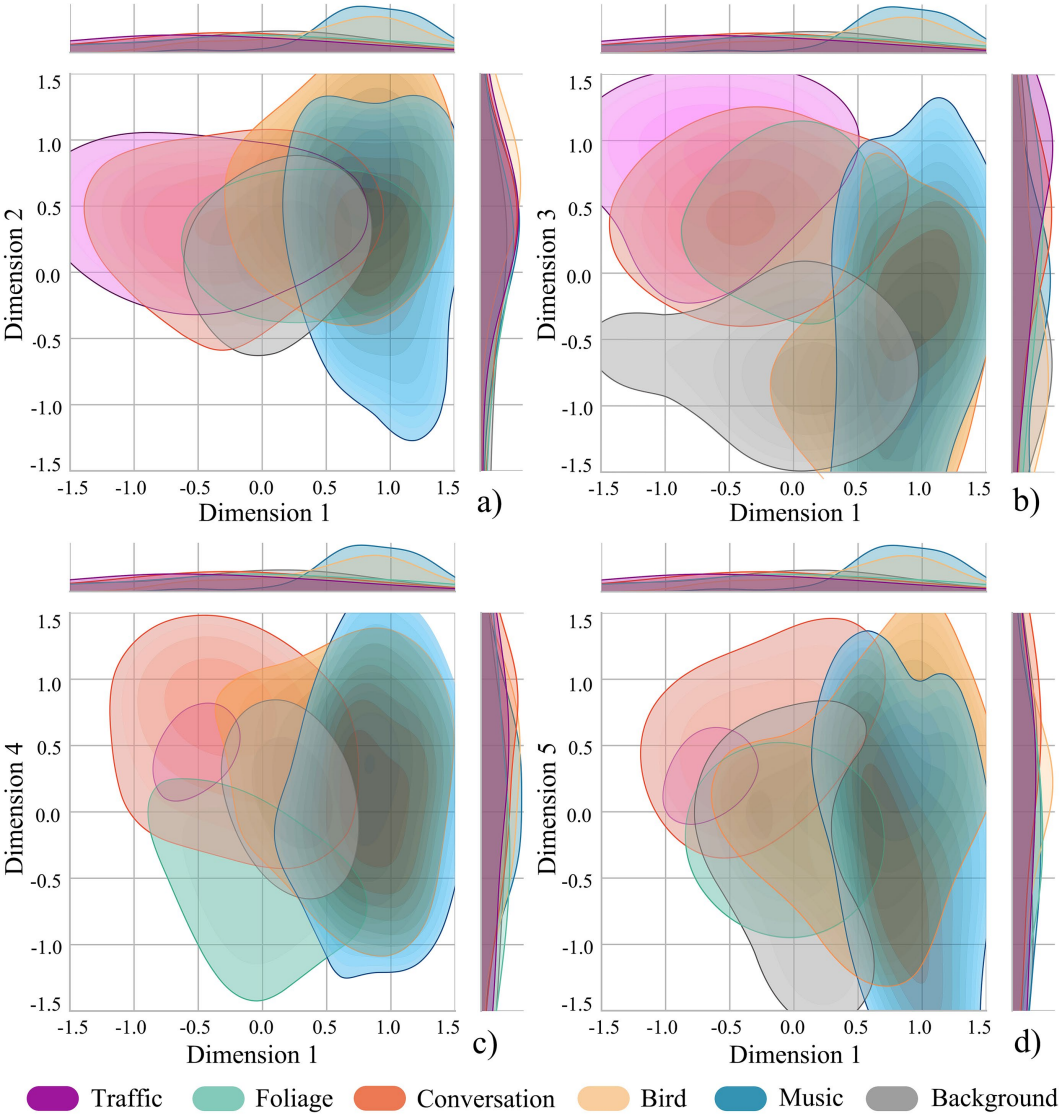
Distributions of factor analysis for auditory environment perception. **(a)** Dimension 1 versus Dimension 2; **(b)** Dimension 1 versus Dimension 3; **(c)** Dimension 1 versus Dimension 4; **(d)** Dimension 1 versus Dimension 5.

The two-dimensional distribution of the factor analysis for auditory environment perception is shown in Figure 4. Figure 4a shows that along Dimension 1, bird sound and music were positioned in the positive region of Dimension 1, indicating that these two sounds scored highly on auditory affect; traffic sound was mostly distributed in the negative region of Dimension 1. Along Dimension 2, the bird sound was positioned in the positive region, indicating a clear distinction in auditory spatiotemporality. The other sounds showed no obvious differentiation along Dimensions 1 and 2. Figure 4b shows that along Dimension 3, traffic, foliage, and conversation sounds were distributed in the positive region, indicating high scores on auditory discriminability for these three sounds, while the background sound was distributed in the negative region of Dimension 3. Figure 4c shows that along Dimension 4, traffic and conversation sounds were mostly distributed in the positive region, indicating high scores on auditory awareness for these two sounds, whereas foliage sound was mostly distributed in the negative region. Figure 4d shows that along Dimension 5, traffic and conversation sounds are distributed in the positive region, indicating high scores on auditory curiosity for these two sounds, whereas other sounds show no obvious differentiation in this dimension.

### 3.3 Factors of olfactory environment perception

**Figure 5:**
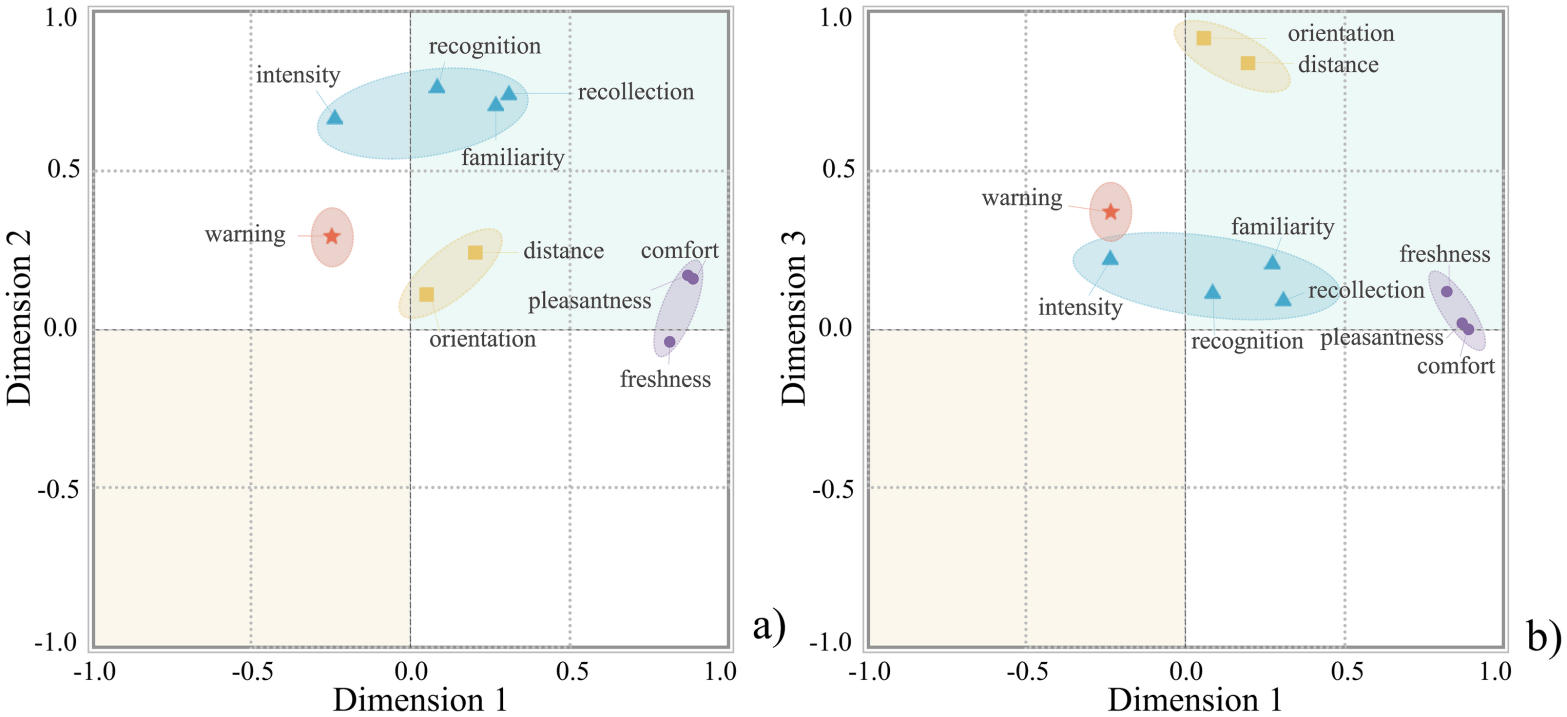
Factor loading plot of olfactory environment perception. **(a)** Dimension 1 versus Dimension 2; **(b)** Dimension 1 versus Dimension 3.

Bartlett’s test of Sphericity for olfactory environment perception was significant (*p* < 0.001) with a KMO value of 0.748, indicating that the data were suitable for factor analysis. The rotated factor matrix of olfactory environment perception is shown in Table 5, and the factor loading plot of olfactory environment perception is presented in Figure 5. Different factors exhibited clear distributions across various dimensions. Factor 1 had high loadings on pleasantness, comfort, and freshness and can be called olfactory affect. Figure 5a shows that all three indicators are distributed along the positive axis of Dimension 1. Factor 2 had high loadings on familiarity, recollection, recognition, and intensity and can be referred to as olfactory awareness. Figure 5a shows that all four indicators are distributed along the positive axis of Dimension 2. Factor 3 had high loadings on distance and orientation and can be referred to as olfactory spatiality. Figure 5b shows that both indicators are distributed along the positive axis of Dimension 3. In contrast, the warning shows low contributions across all three factors and is therefore distributed near the centre across the different dimensions in Figure 5.

**Table 5:** Rotated factor matrix of olfactory environment perception.

Indicator	Factor		
	1	2	3
Pleasantness	**0.869**	0.172	0.019
Comfort	**0.888**	0.167	0.000
Freshness	**0.817**	−0.033	0.119
Familiarity	0.267	**0.706**	0.198
Recollection	0.311	**0.740**	0.087
Recognition	0.083	**0.767**	0.123
Intensity	−0.244	**0.664**	0.221
Warning	−0.246	0.305	0.367
Distance	0.195	0.248	**0.829**
Orientation	0.055	0.113	**0.909**

Bold indicates matrix loadings greater than 0.5.

**Figure 6:**
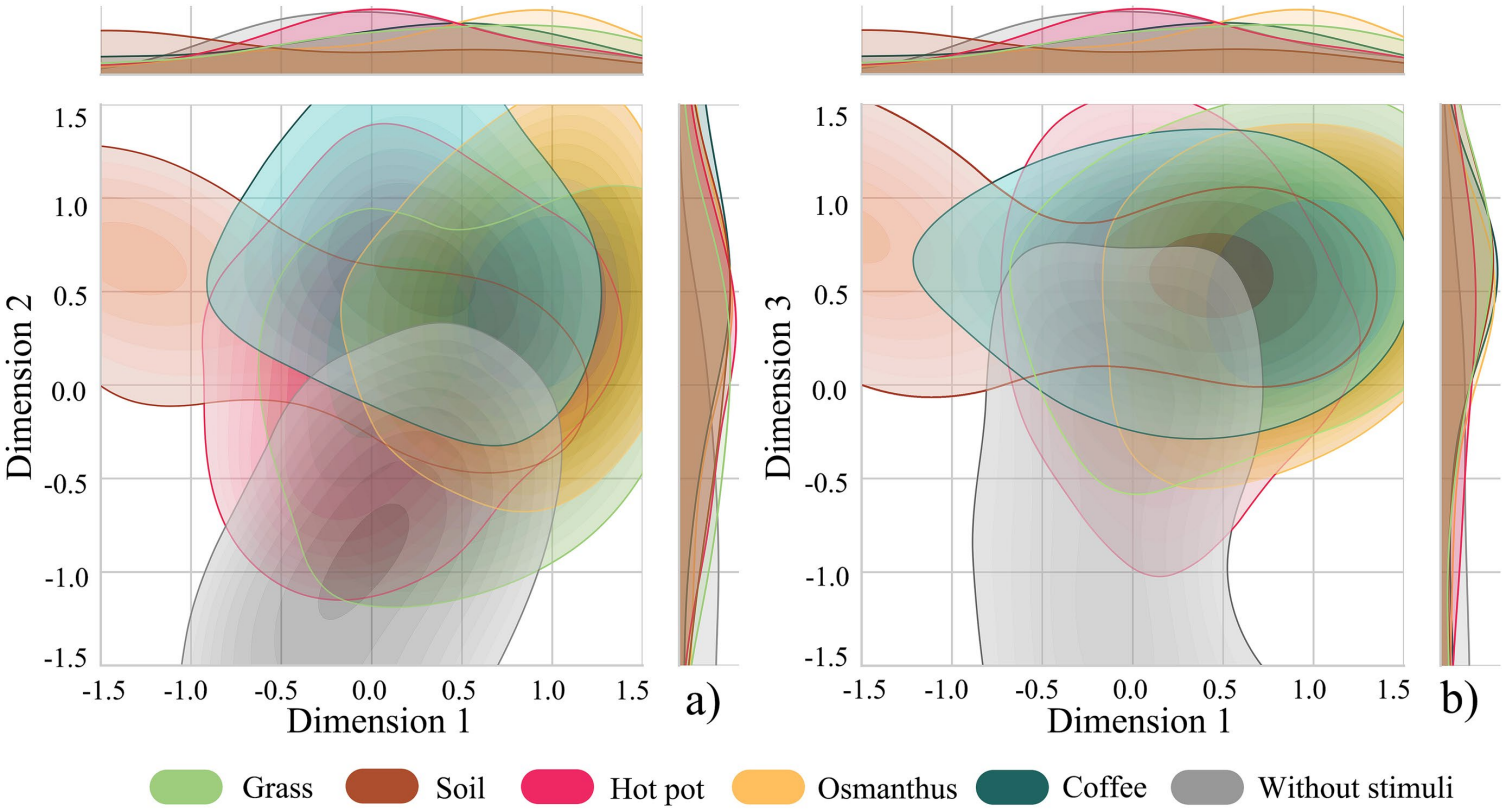
Distributions of factor analysis for olfactory environment perception. **(a)** Dimension 1 versus Dimension 2; **(b)** Dimension 1 versus Dimension 3.

The two-dimensional distribution of the factor analysis for olfactory environment perception is shown in Figure 6. Figure 6a illustrates that along Dimension 1, the grass and osmanthus odours are positioned in the positive region, indicating that these two odours score highly on olfactory affect. Along Dimension 2, the odours of soil, coffee, and osmanthus were mostly located in the positive region, suggesting high scores on olfactory awareness for these three odours, while the without stimuli condition was situated in the negative region of Dimension 2. Hot pot odour did not show a clear differentiation along Dimensions 1 or 2. Figure 6b shows that along Dimension 3, the odours of grass, soil, osmanthus, and coffee were predominantly distributed in the positive region, indicating a high degree of differentiation in olfactory spatiality. The hot pot odour and the condition without stimuli exhibited overlapping distributions across the positive and negative regions, indicating limited differentiation of this factor.

## 4 Discussions

### 4.1 Discussion on simplifying indicators of auditory environment perception

Following the dimensionality reduction of the auditory environment perception, indicators within the same factor may exhibit collinearity owing to semantic similarity. Consequently, a correlation analysis was conducted to explore whether the highly correlated indicators could be streamlined (using a correlation coefficient greater than 0.5 as the threshold). Spearman’s rank correlation analysis revealed that for Factor 1, pleasantness showed a significant positive correlation with comfort (r = 0.742, *p* < 0.001) and safety (r = 0.622, *p* < 0.001), whereas it showed a significant negative correlation with annoyance (r = −0.511, *p* < 0.001) and loudness (r = −0.532, *p* < 0.001). Comfort was significantly positively correlated with safety (r = 0.563, *p* < 0.001) and negatively correlated with annoyance (r = −0.553, *p* < 0.001) and loudness (r = −0.554, *p* < 0.001). A significant negative correlation was also observed between safety and loudness (r = −0.501, *p* < 0.001). Conversely, annoyance was significantly and positively correlated with loudness (r = 0.541, *p* < 0.001). Fear showed no strong correlation with other indicators. This suggests that pleasantness, comfort, safety, annoyance, and loudness share a close intrinsic relationship. Audition is a key sense for environmental perception, navigation, and self-protection among the visually impaired. In most cases, if they feel pleasant and comfortable in an auditory environment, they are more likely to feel safe and not annoyed. Conversely, environments with higher loudness tend to induce more negative effects. Therefore, comfort, pleasantness, and annoyance may be merged, for example, by representing all three through comfort. In previous studies, comfort and pleasantness in the auditory environment were generally not clearly distinguished semantically (Torresin et al., 2023), and one of these indicators was typically selected for the research. However, when investigating the impact of the auditory environment on emotions, the term pleasantness should be used as a professional descriptor (Osgood, 1957; Bradley and Lang, n.d.). Safety (or danger) has also been confirmed in prior research as a unique dimension of soundscape perception for the visually impaired, which relies solely on auditory cues (Mediastika et al., 2020). Loudness, on the other hand, tends to refer to the physical evaluation of sound pressure level. Therefore, despite its strong correlations with the other indicators, it is not advisable to merge it with them. Loudness is commonly regarded together with acoustic comfort as one of the most important indicators in soundscape evaluation (Yu and Kang, 2009). Fear was retained in all cases. For Factor 2, a strong positive correlation was observed between distance and orientation (r = 0.627, *p* < 0.001). When visually impaired individuals rely on auditory cues to construct spatial experience, they first determine the approximate orientation of the sound source and then judge the distance based on the sound’s loudness. Therefore, these two work synergistically in locating objects or people and are both indispensable; hence, they were not considered for merging. For Factors 3 and 4, no significantly strong correlations were found between the different indicators. Factor 5 included only curiosity, which showed no significantly strong correlation with any other indicator. Therefore, the indicators within these three factors could all be retained.

### 4.2 Discussion on simplifying indicators of olfactory environment perception

Based on the correlation analysis of the olfactory indicators for Factor 1, pleasantness showed a strong positive correlation with comfort (r = 0.799, *p* < 0.001) and freshness (r = 0.566, *p* < 0.001), whereas comfort was also strongly correlated with freshness (r = 0.581, *p* < 0.001). This suggests that for visually impaired individuals, a fresh olfactory environment often evokes both pleasantness and comfort. Before considering indicator merging, whether pleasantness and comfort were rated at similar levels in the experiment was examined. Across all olfactory trials, comfort was rated at (M = 3.56, SD = 1.245), whereas pleasantness was rated at (M = 3.50, SD = 1.201). And a paired comparison between pleasantness and comfort ratings was additionally conducted to quantify the magnitude of the difference (*p* = 0.318 > 0.05). These results provide a descriptive basis for the subsequent discussion on whether the two indicators could be streamlined. Therefore, it may be appropriate to consider combining comfort and pleasantness. Previous research has shown that freshness is an important factor influencing pleasantness (Xiao et al., 2018); therefore, the retention of freshness was considered. For Factor 2, familiarity and recollection showed a strong positive correlation (r = 0.510, *p* < 0.001). For the visually impaired, a familiar olfactory environment often evokes related past recollections, both of two indicators involve past experiences; however, they refer to different levels of psychological experience and cognitive processes. The generation of familiarity is believed to result from odour signals transmitted directly from the olfactory bulb to the perirhinal cortex, bypassing the thalamus and rapidly activating emotion-related brain regions (e.g., the amygdala). Recollection (especially episodic recollection), however, strongly relies on the hippocampus and its extensive connections with cortical regions. It is responsible for binding various types of information (time, place, sensory details, emotions) to a coherent memory trace, which can then be retrieved when needed (Fan and Guo, 2005; Aggleton and Brown, 2006). Recollection and recognition showed a significant positive correlation (r = 0.501, *p* < 0.001). From the perspectives of cognitive psychology and neuroscience, recollection and recognition represent two distinct memory retrieval mechanisms. Previous studies have demonstrated that recognition memory is supported by two independent processes: recollection (a threshold process that retrieves contextual details) and familiarity (a continuous signal that reflects item strength without contextual information), and their dissociation has been validated using receiver operating characteristic (ROC) curve analysis (Yonelinas, 2002). In addition, the correlations between intensity and the other indicators were relatively weak, suggesting that the indicators under Factor 2 (recollection, familiarity, and intensity) can be retained. A strong positive correlation was observed between distance and orientation (r = 0.680, *p* < 0.001). This distinction lies in the fact that the former judges the distance of the odour source based on variations in odour concentration, and relies on temporal gradients in concentration. In contrast, the latter refers to the ability to determine the orientation of an odour source by detecting differences in odour concentration between the two nostrils based on stereo olfaction (Porter et al., 2007). Therefore, it was considered appropriate to retain both indicators. The correlation between warnings and other olfactory indicators was relatively weak. Given the critical role of olfactory warnings in alerting the visually impaired to potential hazards and ensuring their personal safety in daily life, this indicator was retained.

### 4.3 Similarities and differences between auditory and olfactory environment perceptions

Integrating the results from Sections 3.2 and 3.3 reveals the commonality in the main perceptual factors between auditory and olfactory environment perceptions. Factor 1 of them were associated with the affect elicited by the auditory and olfactory environments, with pleasantness and comfort demonstrating the highest loadings on Factor 1 for both. This indicates that comfort represents a fundamental and critical need for visually impaired individuals to perceive both urban auditory and olfactory environments. Concurrently, differences were observed between groups. Regarding the auditory affect, in addition to pleasantness and comfort, high loadings were evident for safety, annoyance, fear, and loudness. In contrast, for olfactory environment perception, only freshness retained a notably high load within the olfactory affect factor. This suggests that for the visually impaired, the auditory environment appears to elicit a richer perception of the affect. Furthermore, Factor 2 (auditory spatiotemporality) in auditory environment perception and Factor 3 (olfactory spatiality) in olfactory environment perception shared similar indicators: distance and orientation. From an auditory perspective, these two components are closely tied to spatial hearing processes, whereby orientation reflects directional hearing and spatial attention to sound sources, and distance judgements are supported by level-related cues and reverberation information that help estimate proximity and urgency during wayfinding. If spatial experience is retained as an overarching lens, these two items in olfaction can be interpreted in terms of spatial cognitive processes that support everyday wayfinding: distance judgements may reflect perceived intensity/gradient changes over time as one moves through space, while orientation relates to source localisation and spatial updating. In everyday urban settings, relatively stable odour sources may further function as “olfactory landmarks,” supporting place confirmation and route adjustment even when other cues are limited. Similarly, Factors 4 (auditory awareness) and 2 (olfactory awareness) exhibited comparable indicators: familiarity and recognition. This further underscored the perceptual similarities between auditory and olfactory environments. For auditory awareness, familiarity and recognition may indicate how predictable and interpretable the soundscape is, which can reduce cognitive load and increase situational confidence during repeated travel. To strengthen the interpretability of olfactory awareness, these awareness-related items can also be understood through the notion of smellscape presence—that is, the degree to which an odour becomes salient, noticeable, and cognitively accessible in experience rather than remaining in the background (Torriani et al., 2025). Higher olfactory presence may increase the likelihood that odour cues are encoded and retrieved as part of place experience, thereby supporting place recognition, memory, and confidence during wayfinding.

However, the relative prioritisation of these factors with shared indicators differed across modalities. This indicates that for the visually impaired, the auditory environment provides more comprehensive informational content, encompassing both temporal and spatial dimensions. In contrast, the olfactory environment demonstrates a stronger association with memory and predominantly provides spatial information to contextualise their navigational experience. Factor 3 (auditory discriminability) encompassed the unique auditory perceptions of visually impaired individuals, including reverberation, interference, guidance, and warning. While warning was also identified as an indicator in the olfactory perception interviews, it exhibited a low factor loading in the subsequent factor analysis. This low loading may be attributed to the selection of olfactory stimuli. To comply with ethical standards and avoid causing harm to visually impaired participants, all odour materials used in the experiment were safe and non-toxic and did not include irritating or hazardous odours that might trigger a strong warning response. Consequently, the participants were unlikely to perceive a pronounced sense of warning from the olfactory environment in the experimental setting, leading to a lower loading of this indicator in the data analysis.

### 4.4 Limitations and implications for inclusive and universally accessible design

A key limitation is that odours were simulated in a laboratory using essential oils/perfumes, which cannot fully reproduce real-world smellscapes where odour plumes vary dynamically with airflow mixtures of multiple sources. Laboratory also reduces contextual cues that may shape odour meaning *in situ*, and repeated exposure may introduce adaptation or carry-over despite ventilation intervals. In addition, for ethical and safety reasons this study excluded hazardous or irritating odours (e.g., pollution-related sources), which may partly explain the weaker contribution of “warning” in the olfactory structure. These constraints suggest that the present factor structures should be interpreted as an experimentally tractable subset of everyday auditory–olfactory experience, and future work should validate and refine the model through in-situ studies (e.g., smellwalks/soundwalks in community settings) and, where appropriate, by including a broader range of ecologically valid odour sources. In addition, factor retention in this study was based on the Kaiser criterion together with scree-plot inspection; more stringent procedures such as parallel analysis or the MAP test were not applied additionally. Given the exploratory aim and the grounded-theory-derived indicator set used for initial dimensionality reduction, interpretable criteria within SPSS was prioritised commonly used. Future work could incorporate parallel analysis/MAP as robustness checks. Finally, as the present EFA trial-level observations for exploratory purposes, within-subject dependency and potential within−/between-participant structure differences were not modelled. Given that internal consistency varied across dimensions, more complex approaches (e.g., multilevel factor analysis or clustered confirmatory models) may yield unstable estimates at this stage. Future studies with large-sample research paradigm could apply multilevel factor analysis or clustered (or multilevel) CFA with robust estimation to separately evaluate within- and between-person structures.

For future inclusive and universally accessible design, the identified perceptual dimensions suggest a pathway from perception to design intervention. For audition, enhancing discriminability and directional clarity (e.g., clearer source separation, reduced masking/interference, context-appropriate levels, and consistent spatial/directional cues) may improve orientation confidence and reduce uncertainty during wayfinding. For olfaction, introducing stable, low-intensity, and localised odour cues that are recognisable and non-irritant may function as olfactory landmarks to support place confirmation and route updating. These implications are proposed as testable hypotheses; future studies should verify whether such interventions improve behavioural outcomes (e.g., navigation accuracy, wayfinding efficiency, or hazard avoidance) in ecologically valid settings.

In addition, from a universal design perspective, sensory interventions should support people with visual impairment without imposing undue burden on non-impaired users. For auditory interventions, this implies prioritising informative yet non-intrusive cues (e.g., context-triggered playback, appropriate levels and spectral design) to avoid contributing to noise annoyance. For olfactory interventions, it suggests using low-intensity, localised, and clearly bounded odour sources, avoiding common allergens/irritants, and providing alternative routes or zones for users who prefer to avoid odours. Importantly, co-design and post-occupancy evaluation with diverse user groups can help calibrate these interventions to achieve accessibility benefits while maintaining overall environmental comfort.

## 5 Conclusion

This study aimed to investigate the parallel auditory and olfactory perceptual model of the visually impaired in urban environments based on spatial experience. Through a combination of semi-structured interviews and laboratory experiment, the following conclusions were drawn.

Regarding the indicators of auditory and olfactory environment perceptions, 324 original descriptions and 17 themes were extracted from the interview results on auditory environment perception. 173 original descriptions and 10 themes were identified from the interview results regarding olfactory environment perception. Themes from both parts were subsequently adopted as evaluation indicators of auditory and olfactory environment perceptions in the experiment.

Five main factors were extracted for the auditory environment perception: auditory affect, auditory spatiotemporality, auditory discriminability, auditory awareness, and auditory curiosity. Bird sound and music scored highly on auditory affect; bird sound also scored highly on auditory spatiotemporality; traffic, foliage, and conversation sounds scored highly on auditory discriminability; and traffic and conversation sounds scored highly on both auditory awareness and auditory curiosity.

Three main factors were extracted for olfactory environment perception: olfactory affect, awareness, and spatiality. The odours of grass and osmanthus scored highly on the olfactory affect; the odours of soil, coffee, and osmanthus scored highly on olfactory awareness; the odours of grass, soil, osmanthus, and coffee exhibited high discriminability on olfactory spatiality, whereas the odour of hot pot and without stimuli showed no significant discriminability on this factor.

## Data Availability

The raw data supporting the conclusions of this article will be made available by the authors, without undue reservation.
