# Supplementary material for: Factor-driven urban sensory equity: parallel auditory and olfactory perceptual models of spatial experience in urban environments for people with visual impairment
Source: Front Psychol. 2026 Mar 9;17:1784190. doi: 10.3389/fpsyg.2026.1784190 (PMC13006277; doi:10.3389/fpsyg.2026.1784190)
Supplement: Supplementary file 1 [file Table_1.docx]

Supplementary Material

# Supplementary Table

**Table 1** The full trial-order schedule

| Participant | Trial 1 | Trial 2 | Trial 3 | Trial 4 | Trial 5 | Trial 6 |
| --- | --- | --- | --- | --- | --- | --- |
| P1 | S4+O4 | S1+O5 | S2+O1 | S5+O3 | S6+O6 | S3+O2 |
| P2 | S4+O1 | S2+O2 | S1+O3 | S3+O5 | S6+O4 | S5+O6 |
| P3 | S5+O4 | S2+O5 | S6+O1 | S1+O6 | S3+O3 | S4+O2 |
| P4 | S6+O2 | S2+O3 | S1+O4 | S4+O5 | S5+O1 | S3+O6 |
| P5 | S4+O3 | S2+O6 | S6+O5 | S5+O2 | S1+O1 | S3+O4 |
| P6 | S1+O2 | S3+O1 | S6+O3 | S2+O4 | S4+O6 | S5+O5 |
| P7 | S2+O1 | S4+O4 | S6+O6 | S3+O2 | S1+O5 | S5+O3 |
| P8 | S3+O5 | S5+O6 | S2+O2 | S1+O3 | S4+O1 | S6+O4 |
| P9 | S2+O5 | S6+O1 | S3+O3 | S4+O2 | S1+O6 | S5+O4 |
| P10 | S1+O4 | S4+O5 | S3+O6 | S5+O1 | S2+O3 | S6+O2 |
| P11 | S3+O4 | S4+O3 | S2+O6 | S1+O1 | S5+O2 | S6+O5 |
| P12 | S4+O6 | S2+O4 | S1+O2 | S5+O5 | S6+O3 | S3+O1 |
| P13 | S6+O6 | S3+O2 | S5+O3 | S1+O5 | S4+O4 | S2+O1 |
| P14 | S1+O3 | S4+O1 | S5+O6 | S6+O4 | S3+O5 | S2+O2 |
| P15 | S6+O1 | S5+O4 | S2+O5 | S3+O3 | S4+O2 | S1+O6 |
| P16 | S3+O6 | S1+O4 | S4+O5 | S2+O3 | S6+O2 | S5+O1 |
| P17 | S1+O1 | S3+O4 | S4+O3 | S6+O5 | S2+O6 | S5+O2 |
| P18 | S2+O4 | S1+O2 | S3+O1 | S4+O6 | S5+O5 | S6+O3 |
| P19 | S5+O3 | S2+O1 | S4+O4 | S6+O6 | S3+O2 | S1+O5 |
| P20 | S6+O4 | S1+O3 | S3+O5 | S2+O2 | S5+O6 | S4+O1 |
| P21 | S1+O6 | S4+O2 | S5+O4 | S6+O1 | S2+O5 | S3+O3 |
| P22 | S5+O1 | S3+O6 | S6+O2 | S1+O4 | S4+O5 | S2+O3 |
| P23 | S5+O2 | S1+O1 | S3+O4 | S4+O3 | S6+O5 | S2+O6 |
| P24 | S6+O3 | S5+O5 | S4+O6 | S1+O2 | S3+O1 | S2+O4 |
| P25 | S1+O5 | S6+O6 | S3+O2 | S2+O1 | S5+O3 | S4+O4 |
| P26 | S2+O2 | S6+O4 | S4+O1 | S5+O6 | S1+O3 | S3+O5 |
| P27 | S4+O2 | S3+O3 | S1+O6 | S5+O4 | S6+O1 | S2+O5 |
| P28 | S2+O3 | S6+O2 | S5+O1 | S3+O6 | S1+O4 | S4+O5 |
| P29 | S2+O6 | S6+O5 | S5+O2 | S3+O4 | S4+O3 | S1+O1 |
| P30 | S5+O5 | S6+O3 | S2+O4 | S3+O1 | S1+O2 | S4+O6 |
| P31 | S3+O2 | S5+O3 | S1+O5 | S4+O4 | S2+O1 | S6+O6 |
| P32 | S5+O6 | S3+O5 | S6+O4 | S4+O1 | S2+O2 | S1+O3 |
| P33 | S3+O3 | S1+O6 | S4+O2 | S2+O5 | S5+O4 | S6+O1 |
| P34 | S4+O5 | S5+O1 | S2+O3 | S6+O2 | S3+O6 | S1+O4 |
| P35 | S6+O5 | S5+O2 | S1+O1 | S2+O6 | S3+O4 | S4+O3 |
| P36 | S3+O1 | S4+O6 | S5+O5 | S6+O3 | S2+O4 | S1+O2 |

Note: S means sound type, each S (S1–S6) across trial positions (each appears 6 times in each Trial 1–6), O means odour type, each O (O1–O6) across trial positions (each appears 6 times in each Trial 1–6); each combo Sn+On across trial positions (each of the 36 combos appears exactly once in each Trial 1–6).

**Table 2** Eigenvalues, variance explained, and cumulative variance for the retained factors

| Modality | Factor | Eigenvalue | Variance explained (%) | Cumulative variance (%) |
| --- | --- | --- | --- | --- |
| Auditory (17 themes) | Factor 1 | 3.881 | 22.8 | 22.8 |
|  | Factor 2 | 2.627 | 15.5 | 38.3 |
|  | Factor 3 | 1.934 | 11.4 | 49.7 |
|  | Factor 4 | 1.188 | 7.0 | 56.6 |
|  | Factor 5 | 1.405 | 8.3 | 64.9 |
| Olfactory (10 themes) | Factor 1 | 2.237 | 22.4 | 22.4 |
|  | Factor 2 | 2.550 | 25.5 | 47.9 |
|  | Factor 3 | 1.836 | 18.4 | 66.2 |
